# Discrete Sequential Information Coding: Heteroclinic Cognitive Dynamics

**DOI:** 10.3389/fncom.2018.00073

**Published:** 2018-09-07

**Authors:** Mikhail I. Rabinovich, Pablo Varona

**Affiliations:** ^1^BioCircuits Institute, University of California, San Diego, La Jolla, CA, United States; ^2^Grupo de Neurocomputación Biológica, Departamento de Ingeniería Informática, Escuela Politécnica Superior, Universidad Autónoma de Madrid, Madrid, Spain

**Keywords:** hierarchical cognitive networks, metastable state brain dynamics, heteroclinic binding, information patterns, control of episodic memory retrieval

## Abstract

Discrete sequential information coding is a key mechanism that transforms complex cognitive brain activity into a low-dimensional dynamical process based on the sequential switching among finite numbers of patterns. The storage size of the corresponding process is large because of the permutation capacity as a function of control signals in ensembles of these patterns. Extracting low-dimensional functional dynamics from multiple large-scale neural populations is a central problem both in neuro- and cognitive- sciences. Experimental results in the last decade represent a solid base for the creation of low-dimensional models of different cognitive functions and allow moving toward a dynamical theory of consciousness. We discuss here a methodology to build simple kinetic equations that can be the mathematical skeleton of this theory. Models of the corresponding discrete information processing can be designed using the following dynamical principles: (i) clusterization of the neural activity in space and time and formation of information patterns; (ii) robustness of the sequential dynamics based on heteroclinic chains of metastable clusters; and (iii) sensitivity of such sequential dynamics to intrinsic and external informational signals. We analyze sequential discrete coding based on winnerless competition low-frequency dynamics. Under such dynamics, entrainment, and heteroclinic coordination leads to a large variety of coding regimes that are invariant in time.

## Introduction

There is a wide variety of cognitive model approaches; most of them refer to specific aspects of cognition such as a language, learning, or decision making. Some efforts aim to develop a general theory for consciousness (e.g., see Baars, 1988; Dehaene, 2014; Tononi and Koch, 2015). Here we focus on a basic low-dimensional model that is able to describe several key information aspects underlying a Dynamical Theory of Consciousness and Cognition. To build this model we used three global concepts: (i) information processing and information generation in the brain as a result of winnerless competition of low-frequency (envelope) variables rather than simply the propagation of information, as many connectionist theories assume; (ii) the transient sequential nature of cognitive processes that can be represented by sequential switching between metastable states, which are reproducible and robust against non-controllable perturbations; (iii) sensibility to informational signals in spite of the robustness of the underlying dynamics.

Note that by envelope variables we refer to macroscopic variables that could be related to ensemble or population activity, in other words, variables that are sufficient to describe the collective slow dynamics of the system. Let us discuss a simple ecological and low-dimensional canonical model satisfying the above principles:

(1) $$\begin{array}{l} \tau_{i}\frac{dX_{i}^{l}}{dt}=X_{i}^{l}(\gamma_{i}^{l}+\beta_{i}W_{i}-\left(\sum_{k=1}^{N}\varsigma_{ik}W_{k}\right)\left(\sum_{j=1}^{N}\rho_{ij}^{l}X_{j}^{l}\right)\\ \;-\sum_{m=1}^{L}\sum_{j=1}^{N}\chi_{ij}^{lm}X_{j}^{m}+\xi_{X}^{l}\left(t\right))+q\varphi_{i}\left(\Omega ,t,X_{i}^{l}\right) \end{array}$$

(2) $$\begin{array}{rcl} \mu \frac{dW_{i}^{l}}{dt} & = & W_{i}^{l}\left(\zeta_{i}^{l}+\eta_{i}X_{i}^{l}-\overset{N}{\underset{j=1}{\sum }}\theta_{ij}^{l}W_{i}^{l}+\xi_{W}^{l}\left(t\right)\right) \end{array}$$

Equations (1, 2) represent a cognitive model that, in particular, describes two mutually modulated cognitive processes, for example, autobiographic memory recall and attention focusing, or interaction of cognitive and emotional processes under limited attention. In this model, $X_{i}^{l}$ is the intensity of the *i-*th pattern of cognitive information (e.g., memory). $W_{i}^{l}$ is the sequential intensity of a cognitive resource (e.g., attention and/or emotion). Inhibitory nonsymmetric connection matrices $\rho_{ij}^{l}$, $\theta_{ij}^{l}$, and $\chi_{ij}^{lm}$ provide sequential winnerless competition (WLC), i.e., switching dynamics for the information patterns and the associated cognitive resources (Rabinovich et al., 2001, 2006b, 2008a). $\gamma_{i}^{l}$ and $\zeta_{i}^{l}$ represent the self-excitation, and β_*i*_ and η_*i*_ describe the mutual excitation for each variable. ς_*ik*_ is responsible for cognitive inhibition control. τ_*i*_ ~1, μ < < 1 characterizes fast attention switching, and $\xi_{X}^{l}$, $\xi_{W}^{l}$ represent small noise in the ***X*** and ***W*** dynamics, which is used to discuss the robustness of the transients (see Glossary). The order of the patterns in a sequence is determined by the connection matrices and is invariant to time scaling. The functions $\varphi_{i}\left(\Omega ,t,X_{i}^{l}\right)$ represent a rhythmic external forcing that we will discuss below. This canonical model can be easily generalized to describe several interacting cognitive processes and resources.

We argue that goal-directed functional cognitive activity and also thought generation, imagination, creativity, and emotions are processes that rely on transient sequential brain activities. A large array of cognition related processes can be understood and predicted through the analyses of the temporal switching between different brain network modes that we name information patterns and which can be represented in the model described above by envelope variables. To be robust and sensitive at the same time, the dynamics that describes such patterns has to satisfy a set of rules: (i) winnerless competition between modes, (ii) hierarchical functional organization of the global networks and the cognitive resources, (iii) hierarchical stability of the multilevel architecture (Rabinovich et al., 2012b). To follow these principles in our dynamical models, it is necessary to use the concept of inhibition at all levels: cognition, emotion, metacognition, and behavior. This concept can be generalized to social cognition as well. Inhibitory processes have been postulated to explain decrements or changes in task performance in many domains of psychological research, and experimental evidence shows that such inhibitory processes exist (Aron, 2007; Munakata et al., 2011; Schilling et al., 2014). The architecture of the inhibitory networks and the levels of inhibition are represented in model (1)–(2) by the intrinsic structure of the connection matrixes, which are asymmetric to guaranty the WLC dynamics.

Hierarchical sequential dynamical coding is a key concept for cognitive dynamics. It refers to coding in the form of a hierarchy of sequences where the lowest level contains the minimum information for intelligibility. Succeeding layers of the hierarchy add robustness to the scheme. This concept can be easily illustrated on a language example (Cona and Semenza, 2017), as sequences of letters compound syllables, syllables compound words, words compound sentences, etc. Language, in fact, is a hierarchical sequential process in which auditory and/or visual patterns learned from other individuals or received from the environment are sequentially encoded, processed, and modified for transferring information to other individuals or to our own semantic memory.

The hierarchical sequential segmentation of information into discrete events—patterns—is a fundamental intrinsic feature of brain dynamics. This concept has been used to design top-down explanations for brain activity on the view that the brain infers causes of its sensory input (Kiebel et al., 2009; Friston et al., 2011). In this setting, hierarchal sequential dynamics in general—and stable heteroclinic channels in particular—have been used as the basis of generative models for the Bayesian brain. We discuss here an adequate mathematical approach that is applicable for the description and prediction of consciousness, emotion, and human behavioral activity.

## Discrete representation of information flows. metastable states and stable heteroclinic channels

WLC network activity provides a mechanism for robust discrete sequential coding of cognitive information. For such processing, information meaning and coherence is more important than information quantity. To deal with cognitive information processes, we have to address context–dependent sequential information and goal–dependent information, e.g., perception depends on ongoing cognitive activity and behavior. The coexistence of bottom-up and top-down discrete information sequences (for example between the prefrontal cortex and hippocampus) produces closed functional loops that lead to the generation of new information, i.e., new thoughts or/and new behavior. This can be the origin of autonomous dynamics with new temporal structure, the sort of dynamics required for creativity (Rabinovich et al., 2012a).

Information feedbacks are crucially important for consciousness as it has been shown by studying the importance of top-down projections in recurrent information processing that involves high-order associative cortices for conscious perception (Boly et al., 2011). Because multiple actions usually cannot be performed at the same time due to the lack of cognitive resources, there is competition between multiple brain systems, (e.g., see Daw et al., 2005).

**Figure 1:**
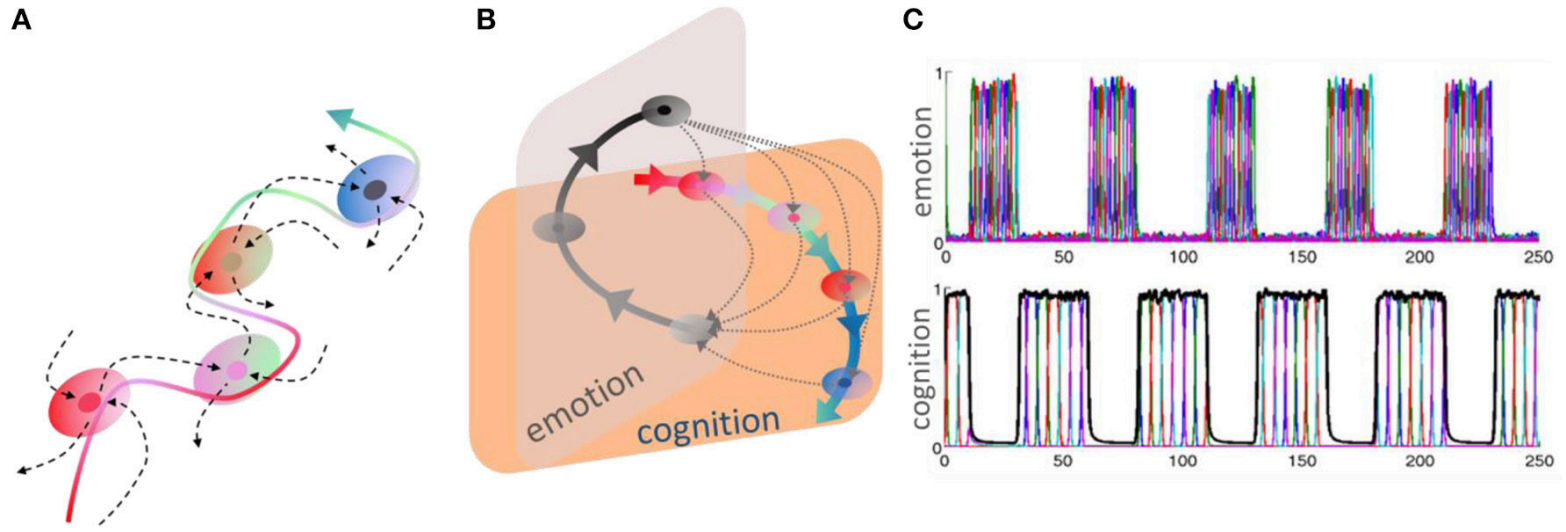
**(A)** Stable heteroclinic channel (SHC), an invariant topological construction. An SHC is a set of metastable states sequentially connected by unstable seperatrices. The robustness of such channel means that trajectories in the neighborhood of the sequence of separatrices do not leave it until the end of the channel is reached (Rabinovich et al., 2012a, 2015). **(B)** This panel shows heteroclinic channels representing a recurrent cognitive-emotion interaction—the dotted trajectories illustrate that the interruption of cognitive performance by emotion, which can happen at any cognitive stage. Adapted from Rabinovich and Varona (2017). **(C)** Time series of sequential switching of emotional and cognitive modalities from model (1)–(2), (Rabinovich et al., 2010b).

Thus, a relevant question here is how to mathematically represent and describe the evolution of cognitive information in time. A stable heteroclinic channel is a convenient mathematical image to describe robust cognitive information flows based on sequential dynamics. It is defined as a sequence of successive metastable (saddle) states in the phase space (Rabinovich et al., 2008b, 2015). These saddles can be pictured as successive and temporary winners in a competitive information scenario (see Figure 1).

Mathematically, a stable heteroclinic channel can be explained as follows. Suppose we have a dynamical model in the form of differential equations:

(3) $$\begin{array}{r} \text{d}\text{x}/\text{dt}=\text{f }\left(\text{x}\right) \end{array}$$

where vector **x** ϵ R^n^. This system gives rise to a heteroclinic sequence if it has a finite sequence {*Q*_1_*, Q*_2_*,…,Q*_*N*_} of equilibrium points, and at *Q*_*i*_ the eigenvalues of the linearization of Equation (3) can be ordered as Rabinovich et al. (2008b):

(4) $$\begin{array}{r} \lambda_{1}^{{\;}^{\left(i\right)}}>0>Re\;\lambda_{2}^{{\;}^{\left(i\right)}}\geq Re\;\lambda_{3}^{{\;}^{\left(i\right)}}\geq \cdot \cdot \cdot \geq Re\;\lambda_{n}^{{\;}^{\left(i\right)}}. \end{array}$$

Thus, each *Q*_*i*_ is a saddle with a one-dimensional unstable manifold—separatrix—which connects each saddle with the next one to form a heteroclinic sequence. When the saddle value ν_*i*_ = –Re λ^(*i*)^_2_ /λ^(*i*)^_1_ for *Q*_*i*_ is positive ν_*i*_ > 1, then the saddle *Q*_*i*_ is called dissipative. In this case, the compression along the stable manifolds dominates the stretching along the unstable manifold. If all saddles in the heteroclinic sequence are dissipative, then the trajectories in their vicinity cannot escape from the sequence, providing the stability. If a system has a Stable Heteroclinic Sequence, then it also has a *Stable Heteroclinic Channel* (SHC) like the one illustrated in Figure 1A (Afraimovich et al., 2011).

In the absence of perturbation, the state vector approaching a saddle node along a stable manifold is indefinitely confined to the neighborhood of the saddle. The exit from the neighborhood of a saddle is only possible under a strong perturbation. The dependence of the *exit time* on the perturbation level was studied in Stone and Holmes (1990). A local stability analysis around a saddle fixed point results in the following relation:

(5) $$\tau^{i}=1/\lambda^{i}{}_{1}\;\ln(1/|\eta |)$$

where τ^*i*^ is the mean time spent in the neighborhood of saddle *Q*_*i*_ (provided that the initial points belong to the stable manifold) and |η| is the level of perturbation. Both values of |η| and λ^*i*^_1_ can be controlled by excitation or the interaction with other information modalities, and thus the temporal characteristics of the sequence can be changed. Importantly, the order of the saddles *Q*_*i*_ is invariant and this is a relevant mathematical mechanism for time compression in episodic memory, as we will argue later.

**Figure 2:**
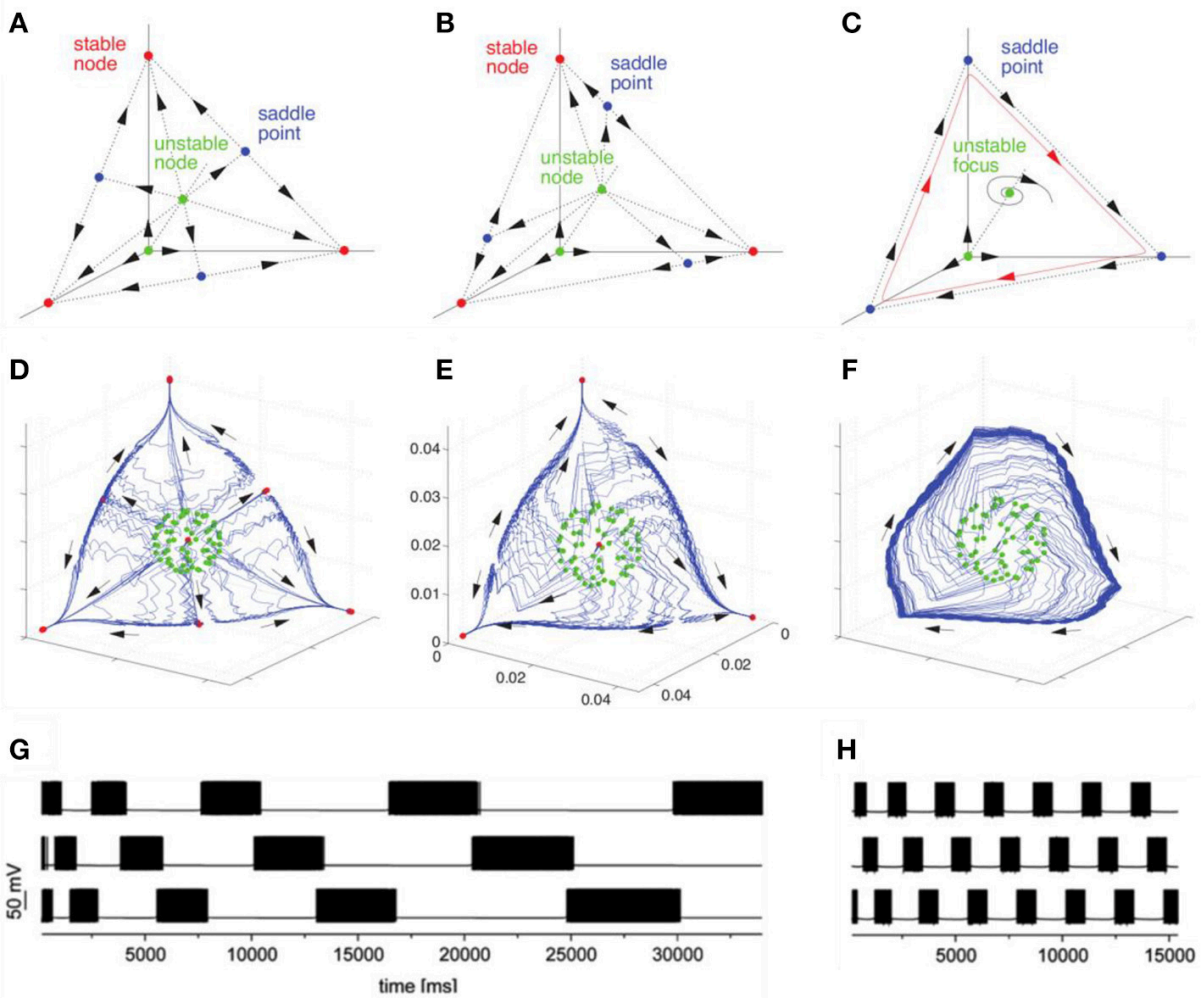
Transition from multistability to WLC dynamics in models with different time scales with connection asymmetry as the control parameter. The figure illustrates the bifurcation toward the birth of a heteroclinic cycle in a Lotka–Volterra model **(A–C)** and in a H–H model **(D–H)**. **(A,D)** represent multistable dynamics (stable fixed points indicated in red correspond to the attractors). **(B)** and **(E)** represent an intermediate case before the annihilation of the stable fixed points (saddles are indicated in blue). **(C,F)** represent the heteroclinic cycle that emerges after the saddle node bifurcation. **(G,H)** represent the time series corresponding to transient heteroclinic dynamics and a robust heteroclinic cycle in the H-H model. Adapted from Nowotny and Rabinovich (2007), Rabinovich and Varona (2011).

The discussed basic model is formulated for the description of neuronal envelope or rate group activity. In principle, it can also be formulated in terms of spiking neuronal ensembles (Nowotny and Rabinovich, 2007), see Figure 2. Both rate- and spiking- canonic models contain in the corresponding phase space a powerful dynamical object for sequential information coding—a SHC which allows representing robust transient coding. The necessary condition for its existence is the presence of non-symmetric reciprocal inhibitory connections between the neural groups that form the specific cognitive modes (Afraimovich et al., 2004; Rabinovich et al., 2008b). Here we hypothesize that fast pulsations do not influence slow envelope dynamics as observed, in particular, in fMRI experiments. Recently, in their effort to understand the brain's coding and its canonical computational motifs, Turkheimer et al. observed the phenomenon of self-similarity dynamics at the micro-, meso-, and macro-scale, and suggested that computational motifs are repeated at increasing spatial and *temporal* scales (Turkheimer et al., 2015). Self-similarity phenomena can be observed in the interaction of different temporal scales in the models represented in Figure 2.

In general, we can hypothesize that the WLC dynamics responsible for the discrete sequential information coding supports many kinds of robust brain activity. Models describing such dynamics can be applied at all levels of temporal and spatial hierarchical organization, from motor and sensory processing to higher-level behavior and cognition.

## Sequences of hierarchically organized information patterns: binding and chunking dynamics

**Figure 3:**
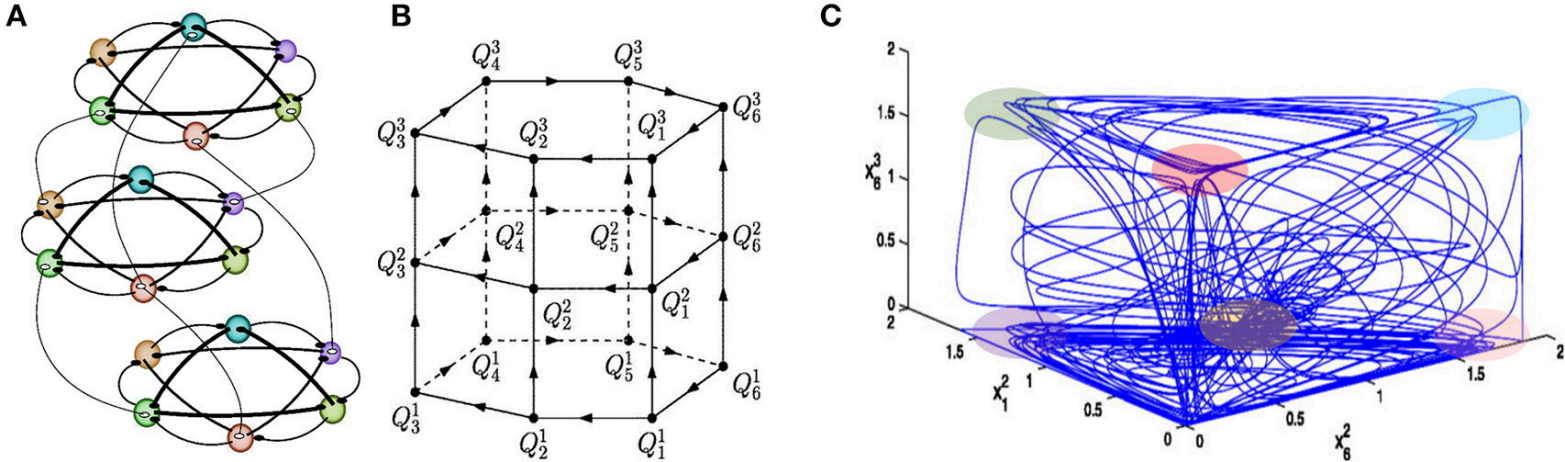
Sequential memory—Binding dynamics of 3-modality events. **(A)** Illustration of an ensemble of 18 competitors fluctuating on three functional communities: each of them is responsible for the processing of different informational modalities. In this example, all connections are inhibitory as characterized by the WLC matrices in the model (1)–(2) with ***W*** = const., i.e., focused attention. **(B)** Illustration of the heteroclinic network in the model phase space, where $Q_{i}^{l}$ is the saddle i in the modality l. **(C)** Mutual modulations of different modalitity sequences, as shown by the projection of a trajectory on a 3-dimensional space. Colored regions point out the vicinity of the metastable states. One can see that this complex trajectory spends some time in a neighborhood of one modality and goes to the next modality afterwards (Afraimovich et al., 2015).

Here we meet the problem of temporal order information coding in memory retrieval in our everyday life. Such retrieval needs binding or association of various features of an event and the preservation of multimodality events in sequential order for all memory types—episodic, semantic, working, etc. Analyses of the robustness of the binding sequential dynamics in the framework of our basic model have illustrated that the recalled sequence can vary depending on intrinsic and environmental conditions (Afraimovich et al., 2015). It has been shown that in the model phase space there exist heteroclinic networks consisting of saddle equilibrium points and heteroclinic trajectories joining them that can bind multi-dimensional events (see Figure 3, Rabinovich et al., 2010a). The binding sequential dynamics is robust for coupled heteroclinic networks: for each collection of successive heteroclinic trajectories inside the joint networks, there is an open set of initial conditions such that the trajectory going through each of them follows the prescribed collection staying in a small neighborhood of it.

The analysis of the complexity and the dependence on the initial conditions in these type of models helps to understand the dynamical origin of well-known cognitive phenomena—episodic memory gaps and errors, i.e., incorrect recalls. In fact, episodic memory can be viewed as a sequential dynamical process that is constructive, rather than reproductive, and it can generate various kinds of errors and illusions, see for example (Schacter and Addis, 2007). Because, as we mentioned above, the heteroclinic sequence of informational patterns is topologically invariant, the SHC is not sensitive to time compressing—allowing to make the time intervals between patterns smaller. This is a possible way to implement time dynamical rescaling and compress time in mind space, specifically in relationship with episodic memory (Howard, 2018).

When we discuss binding, it is necessary to emphasize that functional connections in the human brain are typically more stable within the same modality than across modalities (Zalesky et al., 2014). In general, recent fMRI and electrophysiological studies that have mapped the connections between inter-regional communication and network structure across a diverse range of brain activities demonstrate that the tendency for network reconfiguration depends on behavior (Shine and Poldrack, 2018).

**Figure 4:**
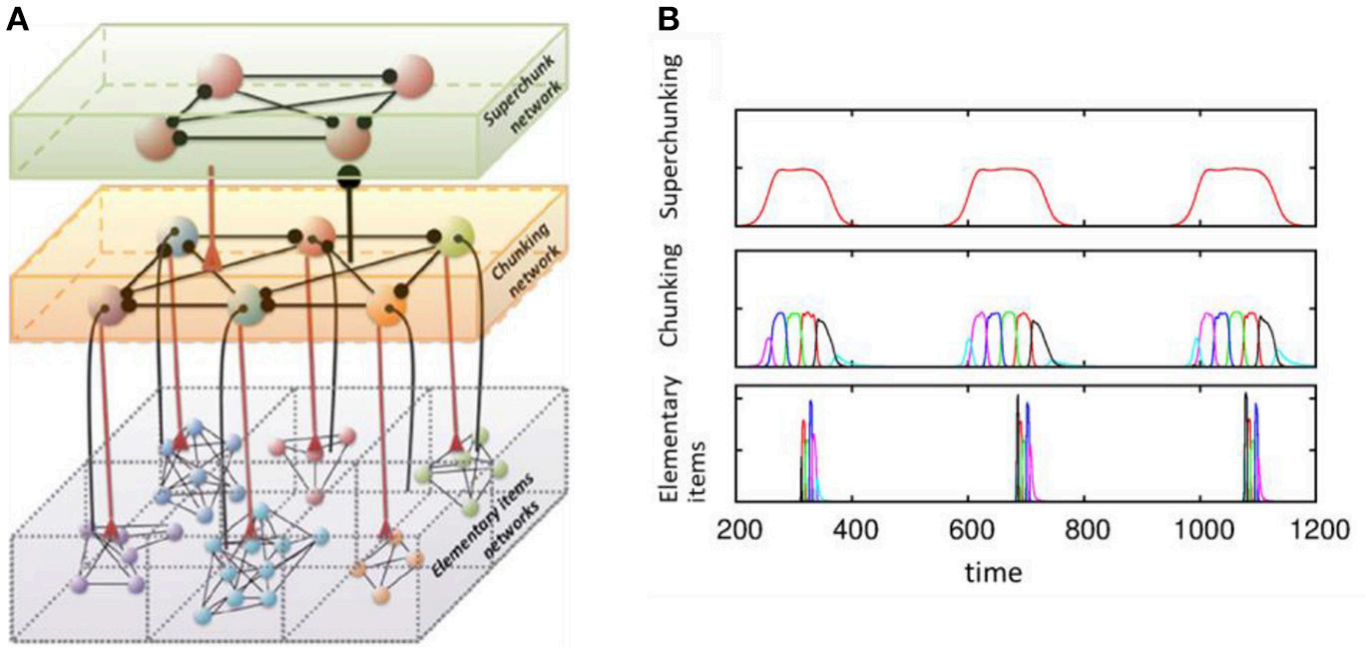
Model of chunking in winnerless competitive networks. **(A)** Illustration of a 3-layer network for hierarchical chunking. **(B**) Time series of the sequences of the three-level hierarchy—items are grouped in chunks; these chunks form 3 superchunks of 6 elements each displaying reproducible dynamics according to the model (1)–(2). Different colors correspond to different items inside each group (switching the color means moving from the previous item to the next one). Adapted from Rabinovich et al. (2014).

Another dynamical cognitive phenomenon that makes use of effective discrete sequential coding is chunking (see Figure 4). Understanding the joint performance of discrete hierarchical cognitive processes is a key part of language processing and behavior programming. The brain solves this problem by grouping information items in a sequence into chunks at different levels of the hierarchy. This can happen on the learning stage, as illustrated in an ecological model displaying heteroclinic dynamics like (1)–(2) (Fonollosa et al., 2015). It has also been illustrated in a system of spiking inhibitory recurrent networks that modeled the mechanisms governing learning in sub-cortical areas (Maffei et al., 2017). Departing from previous modeling results of the striatum (Ponzi and Wickens, 2010), authors have used an anti-Hebbian STDP rule (Fino and Venance, 2010) to demonstrate sequential memory retrieval to control actions.

## Sequential memory and brain oscillations. temporal entrainment and coordination

Recent experiments have demonstrated the key role of low-frequency brain oscillations in information coding. For instance, using optogenetics and fMRI, authors in Chan et al. (2017) discovered robust propagation of low frequency (1 Hz) neural activity, which enhances inter-hemispheric connectivity and mediates sensory processing. Helfrich and Knight in a recent review highlighted several studies that demonstrated that oscillatory dynamics, such as phase resetting, cross-frequency coupling, and entrainment, support the formation of task-relevant coherent functional networks (Helfrich and Knight, 2016). Berens and Horner have discussed experimental findings that provided the first direct evidence that episodic memory formation through binding in humans relies on theta-specific (4 Hz) synchronization mechanisms (Berens and Horner, 2017). Low-frequency oscillations dynamics, in particular synchronization/desynchronization mechanisms, is one of the core phenomena underlying episodic memory formation and reinforcement (Hanslmayr et al., 2016).

It is well known, that our remarkable capacity for language is provided by the combinatorial richness of functional network modes. In a recent paper (Schoffelen et al., 2017), authors showed that communication among language-related areas in the brain is supported by synchronization that forms the modes of the corresponding global networks, see also (Eichenbaum, 2017). Importantly, the different entrainment rhythms reflect the different direction of the information flows. Thus, one can hypothesize that different frequency synchronization phenomena, in fact, control key aspects of the sequential dynamics in global linguistic network architectures. Possibly, it is a generic mechanism for many other cognitive processes that rely on robust sequential activity.

Working memory does not only store information about the items themselves, i.e., the what of the information, but it also dynamically keeps the information about when. Thus, it is a two-modality memory: subject and timing. Recent results show that such information is stored along a logarithmic timeline (Singh et al., 2018).

**Figure 5:**
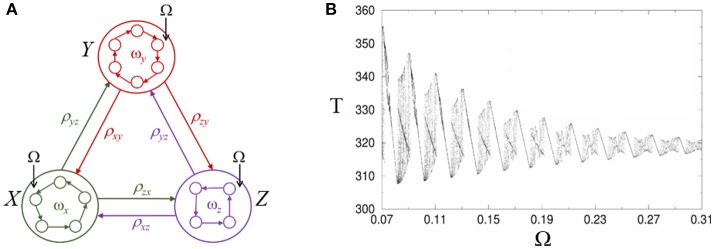
Modulation of retrieval dynamics of episodic memory in heteroclinic networks. **(A)** Networks inside the circles represent the intrinsic architecture of interacting envelope patterns. Ω characterizes the frequency of the external forcing. **(B)** Complex structure of the heteroclinic synchronization: Period T corresponds to the cyclic winnerless competition switching between episodes *X, Y*, and *Z* and is depicted as a function of the forcing frequency Ω under a small Gaussian noise with zero correlation in Equation (1). The region in between synchronized regimes displays chaos (Rabinovich et al., 2006a).

Here we suggest a possible dynamical mechanism for the effective influence of low-frequency oscillations on sequential cognitive processes based on heteroclinic synchronization/chaotization phenomena (Rabinovich et al., 2006a). In Figure 5A we display a heteroclinic network that represents sequential episodic memory, which includes three episodes *X, Y*, and *Z* where each episode or chunk is formed by several events *x*_*i*_*, y*_*i*_, and *z*_*i*_. An external periodic signal with frequency Ω excites each episode trough one of the events, see system (1)–(2). In a general case, the chunk connection matrix ρ_*ab*_ depends on the episode frequency ω_*x, y, z*_.

As one can see in the Figure 5B, the synchronization intervals (with a linear dependence between the period of the chunk episode cyclic switching and the forcing frequency) are separated by intervals with complex dynamics including areas with period-doubling bifurcations and chaos. In a general case, the individual dynamics of different episodes will be different and will distinctly evolve under the action of the periodic forcing. The control frequency Ω leads to a change of the whole episode dynamics and, in fact, is equivalent to a dynamical change of the episodic memory network architecture.

There are several scenarios where rhythmic modulation of cognitive dynamics occurs. For example, rhythmic breathing creates electrical activity in the human brain that enhances emotional judgments and memory recall. Nasal respiration entrains human limbic oscillations and modulates cognitive processes (Zelano et al., 2016). In a different context, it has been shown that specific pieces of music can elicit strong emotions in listeners and, possibly in connection with these emotions, specific memories can be remembered even years later (Eschrich et al., 2008; Jäncke, 2008; Janata, 2009).

**Figure 6:**
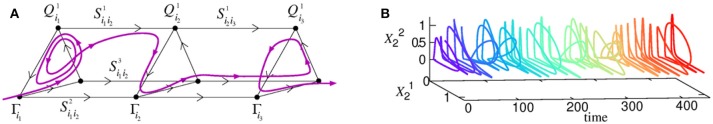
**(A)** An illustration of a 3-modality heteroclinic network and one of the associated trajectories in its vicinity corresponding to the binding process. **(B)** Joint dynamics of two temporally coordinated binded modalities (Rabinovich et al., 2010a). The time series is plotted with a color-code representing the evolution of time to favor the visualization.

Cognitive information processing must contain a mechanism of binding between different information modalities in the brain. Here we consider heteroclinic binding of sequences (Rabinovich et al., 2010a; Varona and Rabinovich, 2016). The basic model (1)–(2) that we propose is able to explain the origin of the temporal coordination of competitive dynamics of active brain modes representing the processing of different cognitive modalities and resources in parallel. The model describes the coordination of spatio-temporal patterns in the form of sequential switching corresponding to different modalities through their dynamical connections, which are represented in the phase space by several unstable separatrices (see Figures 3B, 6). We have previously formulated the conditions for the existence of a multimodality heteroclinic sequence in the phase space of this model (Rabinovich et al., 2010a). Such a sequence appears due to inhibitory connections between different networks that implement a winnerless competition interaction.

**Figure 7:**
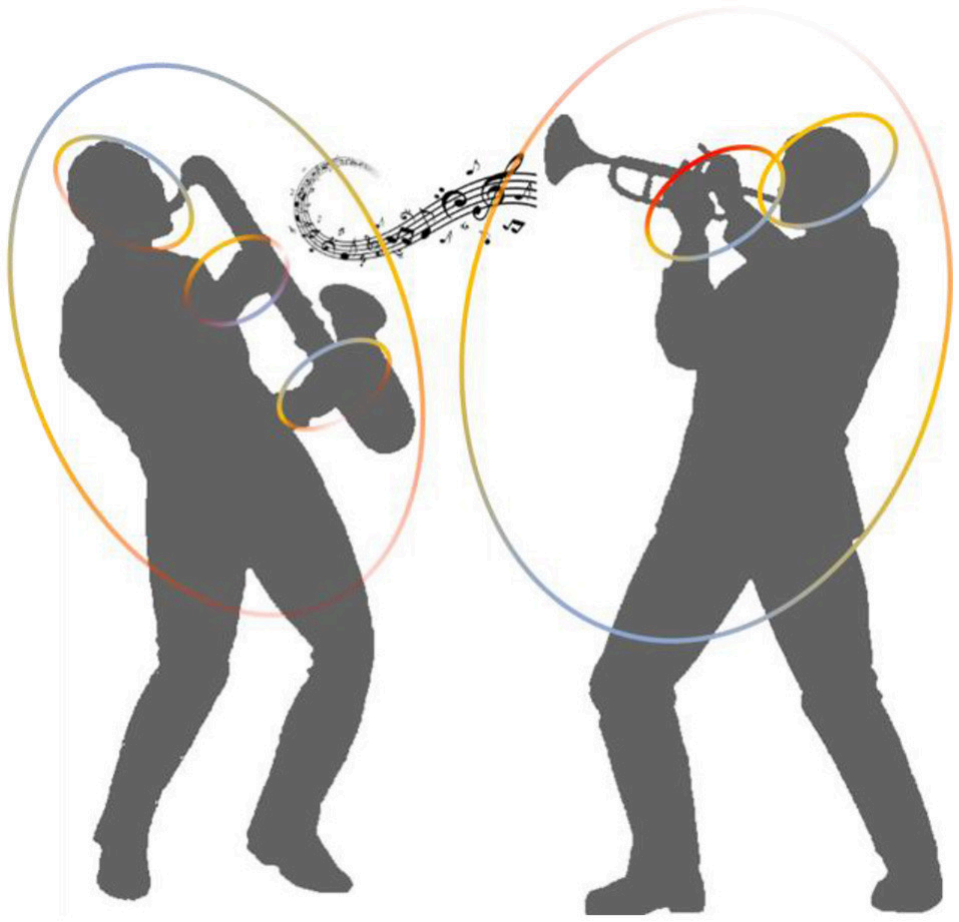
Representation of the visual and auditory sequential information exchange in musical improvisation. Adapted from Walton et al. (2015), (see also Kugler and Turvey, 1987).

Temporal coordination is a key factor in performing all multimodal cognitive human activities. For example, music perception and dance creativity, including emotion as a cognitive resource, requires the temporal binding of all sensory modalities involved. Here, a challenging problem from the theoretical point of view is to understand how sequential imagery, attention and intrinsic rhythms contribute to high precision performance in musical and dance ensembles. The problem becomes even more complex when we try to consider the ability of several musicians to improvise, where they must spontaneously coordinate their sequential actions with co-performers in order to produce novel musical expressions (see Figure 7).

Investigations of such behavior have traditionally focused on describing the creation of cognitive structures and the ability of the time-evolving patterns to perform inter-musician movement coordination. Revealing the mechanisms underlying the coordination of precise movements among improvising musicians is an very important step toward the understanding of how creative musical expressions emerge from the spontaneous coordination of multiple sequential musical bodies (Walton et al., 2015).

It is known, even across different languages, that our brains show similar activity or become “aligned” when we see the same movie or hear the same music (Hasson and Frith, 2016). We can use this neural phenomenon for a universal modeling of sharing memories and knowledge. Let us illustrate it for a minimal social group with two participants keeping in mind two jazz musicians. In this case, the basic dynamical equations (1) will have the form:

(6) $$\begin{array}{r} \tau_{i}^{x}\frac{dx_{i}^{\;}}{dt}=x_{i}^{\;}\left(\sigma_{i}^{\;}\left(S^{x},R^{x}\right)-x_{i}^{\;}-\overset{N}{\underset{j\neq i}{\sum }}\rho_{ij}^{\;}x_{j}^{\;}-q\overset{M}{\underset{s=1}{\sum }}\vartheta_{is}^{\;}y_{s}^{\;}+\xi_{i}^{\;}\left(t\right)\right) \end{array}$$

(7) $$\begin{array}{r} \tau_{k}^{y}\frac{dy_{k}^{\;}}{dt}=y_{k}^{\;}\left(\delta_{k}^{\;}\left(S^{y},R^{y}\right)-y_{k}^{\;}-\overset{M}{\underset{s\neq k}{\sum }}\xi_{ks}^{\;}y_{s}^{\;}-p\overset{N}{\underset{s=1}{\sum }}\eta_{ks}^{\;}x_{s}^{\;}+\xi_{k}^{\;}\left(t\right)\right) \end{array}$$

For simplicity, we do not represent the dynamics of emotion here, which could be added as in equation (2). Here *x*_*i*_ and *y*_*k*_ are the intensity of different “mind musical modes” of participants *X* and *Y*, σ_*i*_ *(S,R)* and γ(*S, R*) are parameters that represent the auditory and visual sensory mode excitation, and parameters *p* and *q* characterize the strength of the two musician mind interaction. In the case when the information exchange between musicians is only mono-directional (*p* < < *q*)—this can happen if *Y*, for example, does not focus her/his attention on *X*'s visual or auditory signals—this model becomes much simpler and the analytical investigation is possible (see Afraimovich et al., 2018).

Cognitive dynamical process can be considered as a sequential switching from one event or mode in the network to another one and so on according to the “winnerless competition” principle. In Afraimovich et al. (2018) authors have proved that in this “master-slave” case a new dynamical object emerges: a non-smooth invariant torus that is an image of the heteroclinic entrainment. The observed bifurcations there demonstrate dynamics with different levels of complexity and also chaos. Symmetric interactions between musicians usually lead to synchronization, see (Walton et al., 2015).

## Discussion and conclusion

In any environment, the human brain perceives continuous streams of information and automatically segments experiences into a set of discrete events or patterns, see for example (Schapiro et al., 2013; Baldassano et al., 2017). Discrete sequential coding supports most aspects of cognitive activity and brain functions. Global network approaches to modeling and analyses of temporal information dynamics predict emergent cognitive metastable states in hierarchical brain networks. Such prediction of the temporal hierarchical organization of large scale brain networks has been analyzed in fMRI experiments (Vidaurre et al., 2017). The authors showed that the transitions between different metastable states are not random and that the corresponding nonrandom sequencing is itself hierarchically organized revealing two metastable states that demonstrate the brain's tendency to cyclic switching.

Discrete sequential dynamics in the brain are also observed in modern EEG experimental studies (see for review Michel and Koenig, 2018). In particular, it has been reported that prototypic EEG microstates occur in a repetitive sequence across time. These states are reliably identified across subjects. Researchers have proposed that such microstates represent the basic building blocks of a chain of spontaneous conscious mental processes, and that their occurrence and temporal dynamics determine the quality of thinking.

Based on the idea of organized series of conscious states, Dehaene and Changeux formulated the neuronal workspace model (Dehaene et al., 1998, 2003; Dehaene and Changeux, 2011) that, in fact, builds upon Baars model (Baars, 1988, 2002, 2005). They posit that workspace neurons from multiple brain areas become spontaneously co-activated and form discrete spatio-temporal patterns of global activity. Only one such episode of coherent activity is thought to occur at any given moment, i.e., episodes are separated by sharp transitions. In our view, consciousness itself can be parceled into sequential episodes or chunks represented by complex metastable states that form hierarchical heteroclinic networks in the mind space (Rabinovich et al., 2008b, 2015). From a neurophysiological perspective, the robustness of such sequential processes is based on the inhibitory interaction of spontaneously excited metastable states (see also Meehan and Bressler, 2012).

The principles and models that we discussed above can be applied in a wide variety of areas in cognitive science. One of them is language generation and processing. The discrete coding of speech as a coding of sequential thinking is based on “event cell” networks in the hippocampus that can sequentially organize memory for events in temporal order as well as in places (Terada et al., 2017). Linguistic sentences unfold sequentially like a chain of words along time; the underlying syntactic structure can be more complex, in particular, hierarchically organized, and remind a tree of phrases using both mechanisms, i.e., binding and chunking (Nelson et al., 2017).

Finally, we wish to emphasize one more time that discrete sequential information coding is a key mechanism that allows transforming complex cognitive activity into low-dimensional dynamical processes based on the sequential switching between finite (moderate) numbers of patterns or metastable states. The capacity of the corresponding information process can be very large because of the available permutations in ensembles of these states (Rabinovich et al., 2001). Extracting low-dimensional dynamics from multiple large-scale neural populations is currently a hot topic both in cognitive- and neuro- science studies (Gao and Ganguli, 2015; Schneidman, 2016; Nonnenmacher et al., 2017), and will also impact artificial cognitive system approaches. In general, results in this area over the last decade represent a solid base for the creation of low-dimensional models of many types of cognitive functions and allow moving toward a dynamical theory of consciousness.

We would like to end with a remark on the popular view that brain computational models need to be extremely high dimensional to be predictive. This view is based on the fallacy that computational dimension is related to the complexity of the brain itself as a “hardware” system with different interacting spatial scales from which cognition emerge^1^. Such modeling is unfeasible yet, as the brain remains only partially observable. However, we may not need it to explain key aspects of cognitive processes because we are talking about mind dynamics with finite resources, i.e., specific kinds of brain activity such as attention, memory retrieval, decision making, etc. A top-down mathematical model of such processes can be built using the following dynamical principles that we discussed above: (i) clusterization the neural activity in space and time and formation of information patterns; (ii) discrete sequential information coding; (iii) robust sequential coordinated dynamics based on heteroclinic chains of metastable clusters; and (iv) sensitivity of such sequential dynamics to intrinsic and external informational signals. These principles open a new direction for the understanding of the observed brain dynamics and the creation of the basis of a mathematical theory of consciousness.

## Author contributions

MR and PV designed the hypothesis, theory and model. MR and PV wrote the paper. Both authors approved the final version for publication.

### Conflict of interest statement

The authors declare that the research was conducted in the absence of any commercial or financial relationships that could be construed as a potential conflict of interest.

## Footnotes

^1^This popular view is not shared by scientists who subscribe to the Bayesian brain hypothesis or any form of inference. This follows from the fact that in maximizing Bayesian model evidence inherently maximizes the difference between accuracy and complexity. This means that a Bayes optimal dynamics necessarily requires the least complicated, lowest dimensional explanation for high dimensional data.

## Glossary

**Binding**: The process of combining informational features from different sources into one unified object. Heteroclinic binding is a phenomenon represented in phase space by a heteroclinic network formed by metastable states and multi-dimensional unstable separatrices linking them.

**Chunking**: The reduction of hierarchical complexity through the subgrouping of similar proximal pieces of information into singular units to allow further processing. Heteroclinic chunking is a process realized within multiple time scale heteroclinic dynamics.

**Dissipative dynamical system**: If a system is closed, it does not exchange flows of energy, mass, information, etc. with the environment, and the intrinsic volume of the flow is preserved in the phase space. A system with internal friction, inhibition, or radiation is called a dissipative system. In such open systems, the volume of the flow contracts in the phase space. When time goes to infinity, the activity of this system can be represented by attractor dynamics. Dynamical models of cognition as the ones discussed in this paper are exclusively dissipative systems.

**Metastability**: In a metastable state, the variables of a dynamical system reach and temporarily hold stationary values. This is characterized by a slowing down of the motion in the vicinity of the stationary state. On the time series, this phenomenon is represented by a plateau or pause. The image of a metastable state in the phase space is a saddle point and its neighborhood.

**Multistability**: The presence of multiple stable attractors in the phase space of a dissipative dynamical system with their basins of attraction. Such a system serves as a map from the given initial state to an attractor and can be a good model for an associative memory.

**Phase space of a dynamical system**: A space in which all possible states of the system are represented. Each possible state of the system corresponds to one point in the phase space, and close points in the phase space represent close system states. The system evolving over time forms a phase space trajectory. As a whole, the phase portrait represents all behaviors that the system can demonstrate.

**Robust transients**: Trajectories in a phase space of a dynamical model that are disposed in the vicinity of each other when initial conditions are varied. These trajectories are robust against noise (Ventsel and Freidlin, 1970; Freidlin and Wentzell, 2012). Examples of such transients are the trajectories inside a stable heteroclinic channel.

**Stable heteroclinic channel (SHC)**: A transient attractor formed by a sequence of saddle states and their vicinity. If the compressing of the phase volume around the SHC is stronger than the stretching of the volume along the SHC, the trajectories that are attracted by the SHC cannot leave it. SHC denotes the image of robust transient behavior in a dynamical system.

**Winnerless competition (WLC)**: A general dynamical phenomenon that denotes sequential switching of prevalence among participants. For example, if in a head-to-head competition, boxer A beats boxer B, boxer B beats boxer C, and finally boxer C beats boxer A, all participants are “winners” for a finite time, but there is no overall winner such as in “winner takes all.
